# Frailty as an independent risk factor for sepsis-associated delirium: a cohort study of 11,740 older adult ICU patients

**DOI:** 10.1007/s40520-025-02956-2

**Published:** 2025-02-27

**Authors:** Guoqiang Zheng, Jiajian Yan, Wanyue Li, Zhuoming Chen

**Affiliations:** 1 Department of Rehabilitation, The First Affiliated Hospital of Jinan University, Guangzhou, 510630 China; 2 Department of Rehabilitation, Henan Provincial People’s Hospital, People’s Hospital of Zhengzhou University, Zhengzhou, China

**Keywords:** Frailty, Sepsis-associated delirium, Older adults, Sepsis, Intensive care unit

## Abstract

**Background:**

Sepsis-associated delirium (SAD) is a common complication in intensive care unit (ICU) patients and is associated with increased mortality. Frailty, characterized by diminished physiological reserves, may influence the development of SAD, but this relationship remains poorly understood.

**Aims:**

To comprehensively analyze the assessment of frailty as a predictive factor for sepsis-associated delirium in older adults.

**Methods:**

A retrospective cohort analysis was performed on sepsis patients aged ≥ 65 years admitted to the ICU. Frailty was assessed using the Modified Frailty Index based on 11 items including comorbidities and functional status. Patients were categorized into non-frail (MFI: 0–2) and frail (MFI ≥ 3) groups. Delirium was assessed using the ICU Confusion Assessment Method (CAM-ICU) and retrospective nursing notes. Logistic regression analysis was used to examine the relationship between frailty in older patients and the risk of delirium, and odds ratios (OR) and their 95% confidence intervals (CI) were calculated.

**Results:**

Among 11,740 patients (median age approximately 76 years [interquartile range: 70.47–83.14], 44.3% female), frail patients tended to have longer ICU stays, higher severity scores, and potentially worse clinical outcomes. The study found a significant positive association between MFI and the risk of developing SAD (OR: 1.13, 95% CI: 1.09–1.17, *p* < 0.001). Additionally, frail patients had a higher risk of developing SAD compared to non-frail patients (OR: 1.31, 95% CI: 1.20–1.43, *p* < 0.001).

**Conclusions:**

Frailty independently predicts SAD development in older adults with sepsis in the ICU, emphasizing the importance of early recognition and prevention.

**Supplementary Information:**

The online version contains supplementary material available at 10.1007/s40520-025-02956-2.

## Introduction

Sepsis remains a significant global health problem, contributing to approximately 20% of deaths worldwide, and is characterized by life-threatening organ dysfunction caused by an uncontrolled host response to infection [1–3]. This complex syndrome is characterized by a variety of inflammatory responses that can lead to multiple organ failure, with the brain being particularly susceptible [4]. Among the various neurological complications associated with sepsis, sepsis-associated delirium (SAD) has emerged as a common and severe clinical manifestation, occurring in 9-71% of sepsis patients, typically in the early stages of the illness [5, 6]. SAD is defined by acute brain dysfunction resulting from a complex interaction of pathophysiological mechanisms including neuroinflammation, insufficient cerebral perfusion, blood-brain barrier disruption, and neurotransmitter imbalance [7]. The presence of SAD is not a transient complication; it is associated with increased mortality, prolonged hospitalization, and persistent cognitive impairment in survivors [5, 6, 8]. These serious consequences underscore the critical importance of early prevention, timely diagnosis, and effective treatment of SAD by healthcare professionals.

In recent years, frailty has garnered significant attention in critical care medicine, particularly regarding older adult patients in intensive care settings [9]. Frailty is described as a clinical condition characterized by a gradual decline in physiological reserves across several organ systems, leading to heightened susceptibility to acute stressors [10]. This multidimensional construct encompasses physical, cognitive, and psychosocial domains, making it a potentially valuable predictor of adverse outcomes in critically ill patients [11, 12]. Assessment of frailty in critical care has revealed its substantial impact on adverse outcomes among older adults in the Intensive Care Unit (ICU) [13, 14]. Research evidence demonstrates that frail older adults admitted to the ICU face higher risks of prolonged hospitalization, increased complications, and mortality [15–18]. These findings indicate that frailty could be a crucial prognostic factor in the ICU, potentially impacting clinical decision-making and the allocation of resources.

Given the high clinical prevalence and serious consequences of SAD, along with the growing recognition of frailty’s impact on ICU outcomes [19–21], there is an urgent need to elucidate the potential association between these two critical factors. Identification of effective predictors or risk factors for SAD may significantly improve early diagnosis and intervention strategies, potentially reducing associated mortality and improving long-term outcomes for sepsis survivors. In the context of a globally aging population, the study of frailty as a potential risk factor for SAD is particularly important. As the proportion of older adults in the ICU continues to rise and more older adults require intensive care [22], understanding the relationship between age-related vulnerabilities like frailty and acute complications such as SAD becomes crucial for optimizing patient care and resource utilization.

Therefore, this study aims to investigate whether frailty is associated with an increased risk of SAD in ICU patients with sepsis. We hypothesize that frail ICU patients with sepsis have a higher likelihood of developing SAD compared to non-frail patients. By studying this relationship, our goal is to identify high-risk patients who may benefit from enhanced monitoring or preventive measures. This will ultimately improve patient outcomes and optimize healthcare resource allocation.

## Methods

### Study design, setting and population

This study is a retrospective cohort study following the Strengthening the Reporting of Observational Studies in Epidemiology (STROBE) guidelines [23], based on data from the Medical Information Mart for Intensive Care IV (MIMIC-IV) database. The MIMIC-IV dataset, originating from Beth Israel Deaconess Medical Center in Boston, covers comprehensive records from the intensive care units (ICUs) between 2008 and 2019 [24]. The database encompasses a wide range of patient information, including demographics, laboratory test results, nursing observations, disease diagnoses, medication histories, and other basic health data. To ensure the reliability of the study findings and effectively protect patient privacy, the research team rigorously conducted quality control and de-identification processes. All personnel involved in data handling underwent mandatory training and obtained relevant certifications before accessing the database, ensuring their capability in data handling and research ethics. The study strictly adheres to the ethical principles outlined in the Helsinki Declaration, and due to its retrospective nature and lack of direct impact on patient clinical care, individual patient informed consent was not required.

**Fig. 1:**
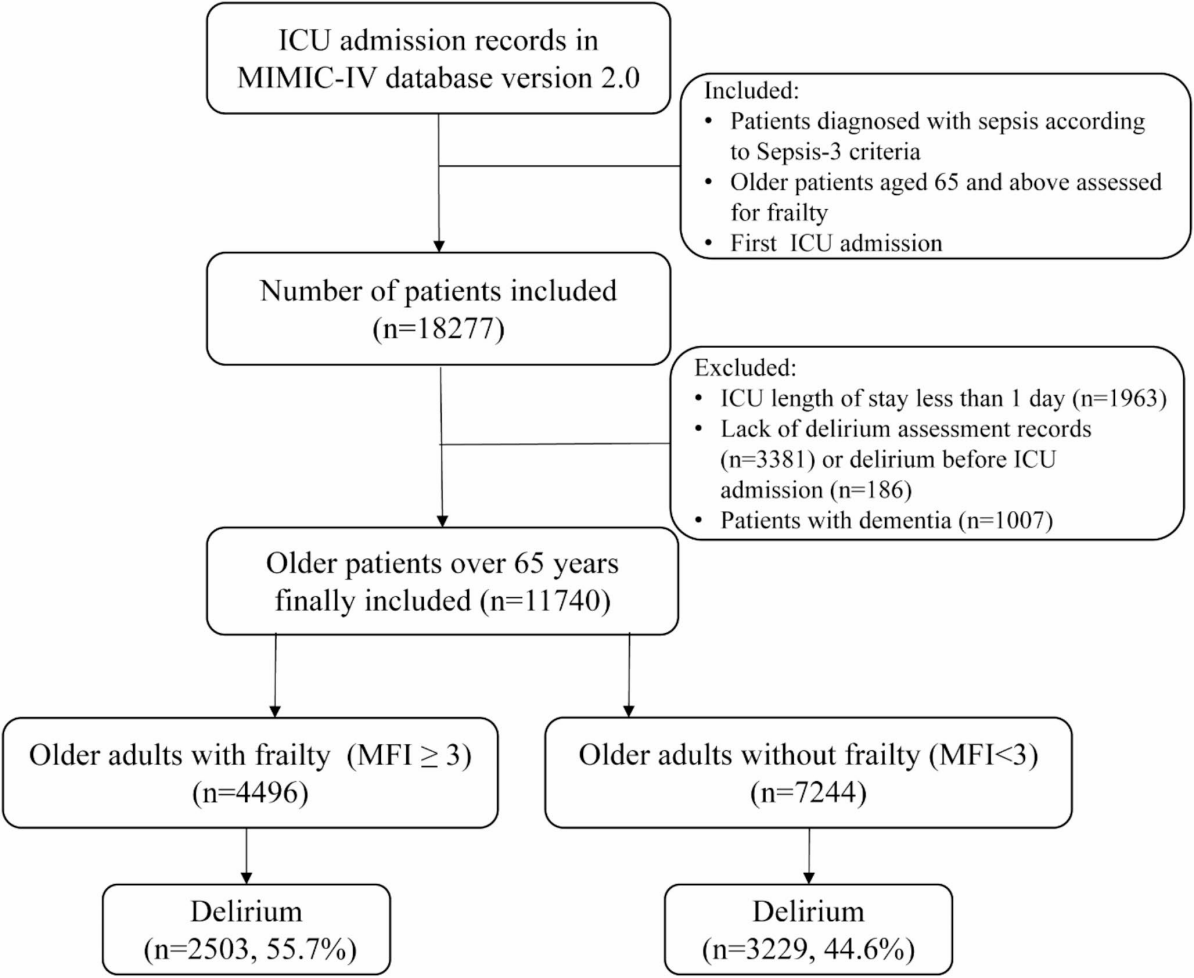
Flowchart of Patient Inclusion and Exclusion Criteria in the Study. Abbreviations: ICU, Intensive Care Unit; MIMIC-IV, Medical Information Mart for Intensive Care IV; MFI, Modified Frailty Index

Study inclusion criteria were: (1) sepsis diagnosis based on Sepsis-3 criteria, defined as host dysregulated response to infection causing life-threatening organ dysfunction, with confirmed or suspected infection and an increase in baseline SOFA score ≥ 2 points2^2^; (2) aged 65 years or older; (3) first admission to the ICU. Exclusion criteria included: (1) ICU stay less than 1 day; (2) absence of delirium assessment or documentation, or presence of delirium prior to admission; (3) presence of dementia [25]. Following these criteria, we identified 11,740 eligible older adults with critical illness and sepsis for further analysis. The patient selection process is shown in Fig. 1.

### Data collection

This study utilized data from the MIMIC-IV database, which has been processed by healthcare professionals to ensure accurate collection and secure storage of patient information [24]. During the data extraction phase, we used Navicat Premium software version 16.0.11 and used its built-in Structured Query Language (SQL) to extract the required data. The data collected includes demographic details, disease severity assessments, vital signs, laboratory findings, complications, treatment plans, medication administration, and adverse effects. Initial measurements of vital signs, laboratory findings, and disease severity assessments were taken upon ICU admission. Comorbidities were identified using International Classification of Diseases, Ninth and Tenth Revisions (ICD-9 and ICD-10) codes [26]. In this study, the missing data rates for all variables were below 20%. Multiple imputation was conducted using the “mice” package, leveraging available variables for training and employing a random forest approach to accurately estimate and impute missing data [27]. Survival duration was calculated from ICU admission to death, with all patients in the MIMIC-IV database having a minimum one-year follow-up period.

### Assessment of frailty

This observational cohort study used frailty as the exposure variable and assessed it using the Modified Frailty Index (MFI) [28]. The revised MFI comprises 11 items, including factors such as diabetes, congestive heart failure, and cerebrovascular events, collectively evaluating patients’ physical function, comorbidities, and physiological impairments. Details of the MFI’s composition and structural classification are provided in Supplementary Table 1. Diagnostic data from the MIMIC-IV database was extracted using ICD-9 and ICD-10 codes, and mapped to corresponding ICD-9 codes in the MFI using a general equivalence mapping from the Centers for Medicare and Medicaid Services (https://www.cms.gov), following Hao et al.‘s approach [29]. These codes facilitated retrieval of relevant MFI components from the database. Each component in the MFI was assigned 1 point, with cumulative scores ranging from 0 to 11. Initially, patients were categorized into three groups based on MFI scores: non-frail (MFI = 0), pre-frail (MFI = 1–2), and frail (MFI ≥ 3) [30]. For analytical clarity, the non-frail and pre-frail groups were subsequently combined into a single ‘non-frail’ category [31].

### Outcomes

The primary outcome was delirium, assessed using the ICU Confusion Assessment Method (CAM-ICU) [32]. Sedation levels were first evaluated using the Richmond Agitation-Sedation Scale (RASS). If the RASS score indicates deep sedation or unarousable state (-4 or -5), assessment cannot proceed. A RASS score equal to or greater than − 3 indicates lighter sedation, necessitating CAM-ICU assessment for diagnosing delirium. CAM-ICU assesses four primary criteria: (1) sudden onset or fluctuating mental status, (2) inattention, (3) changes in consciousness level, and (4) disorganized thinking. Delirium is confirmed when specific combinations of these features are present. Diagnosis of delirium is made if the patient exhibits feature 1 and 2 plus either feature 3 or 4. CAM-ICU, widely adopted by non-psychiatric healthcare providers for its speed, effectiveness, and reliability, shows high sensitivity (84%) and specificity (95%) based on a meta-analysis [33], with nursing notes augmenting diagnostic capabilities by documenting terms such as “delirium,” “confusion,” “agitation,” and “altered mental status” to aid in identifying potential cases of delirium [34, 35].

### Covariates

Covariates are independent variables that may influence delirium development but are not the primary exposure of interest. A random forest algorithm was used to assess the importance of potential predictor variables (Supplementary Fig. 1). This method effectively handles large variable sets, nonlinear relationships, interactions, outliers, and mixed data types, providing reliable variable importance rankings [36]. Upon reviewing the importance scores from the random forest analysis, we observed that significant features were predominantly ranked at scores of 200 and above. Consequently, we opted to focus on variables with importance scores exceeding 200 as primary covariates. These include the Glasgow Coma Scale (GCS), Acute Physiology Score III (APSIII), Sequential Organ Failure Assessment (SOFA), temperature, platelet count, age, weight, mechanical ventilation, mean blood pressure (MBP), red blood cell count (RBC), white blood cell count (WBC), albumin, heart rate, hemoglobin, lactate, respiratory rate, and Braden scale. To ensure the inclusion of clinically meaningful predictors in the model, we consulted with clinical psychiatric professionals and conducted a thorough review of pertinent literature [37–39]. We also included demographic characteristics (sex, race) and clinically relevant variables for delirium (cerebrovascular disease, history of falls, sedative use), despite their lower importance scores in the random forest analysis.

### Statistical analysis

Appropriate statistical methods were used to describe and compare differences between groups. The Shapiro-Wilk test was used to assess the normality of continuous variables, showing that most did not adhere to a normal distribution. Therefore, continuous variables were summarized using median and interquartile range (IQR), while categorical variables were presented as frequencies and percentages. The Wilcoxon rank-sum test assessed group differences in continuous variables, while the chi-square test examined associations in categorical variables. Logistic regression was employed to explore the relationship between frailty and SAD, assessing odds ratios (OR) and corresponding 95% confidence intervals (CI). Variance inflation factors (VIF) were computed to assess multicollinearity in the model, with all variables showing VIF < 4, indicating no significant multicollinearity.

Kaplan-Meier (KM) survival curves were used to evaluate survival rates of patients with and without SAD at 30, 90, 180, and 360 days, with log-rank tests performed to evaluate disparities in survival outcomes between groups. Subgroup analyses were conducted to investigate how demographic and clinical characteristics, such as age (≤ 75 years/>75 years), gender, race (White/other), cerebrovascular disease, myocardial infarction, diabetes, and chronic lung disease, relate to both frailty and SAD.

Statistical analyses were performed using R software (version 4.3.0, https://www.r-project.org/), with a significance level set at *p* < 0.05.

### Sensitivity analysis

To strengthen the validity of our study findings, we undertook several sensitivity analyses. Initially, we employed propensity score matching (PSM) to minimize selection bias and adjust for confounding variables. We constructed a logistic regression model to estimate the likelihood of each individual receiving the intervention or exposure, thereby deriving propensity scores. The model included 22 covariates covering demographic characteristics, severity of illness, cerebrovascular disease, and other factors typical in multivariable models. PSM utilized optimal matching with a 1:1 ratio. Additionally, we applied the Inverse Probability of Treatment Weighting (IPTW) method to assess the relationship between the exposure variable and outcome measures. IPTW is a widely recognized statistical technique in observational studies that aims to mitigate selection bias by assigning specific weights to each subject, creating a pseudo-random sample. The covariates used in IPTW were consistent with those used in PSM. Following PSM and IPTW, we calculated the standardized mean difference (SMD) to assess baseline characteristic balance between matched groups. Typically, an SMD < 0.1 indicates satisfactory balance.

To gauge how unmeasured variables might affect study outcomes, we computed the e-value to assess their potential impact. The e-value, introduced by VanderWeele et al., evaluates how strong an unmeasured confounder would need to be associated with both the exposure and outcome to explain the observed results [40]. A higher e-value suggests greater robustness of study conclusions, indicating less susceptibility to significant influence from unmeasured confounders.

Given that patients who died in-hospital and severe sepsis/septic shock patients typically have more severe conditions and are more prone to delirium, this study conducted sensitivity analysis by narrowing the study population to include only in-hospital survivors (*n* = 9701) and non-severe sepsis/septic shock patients (*n* = 8812). This was done to further explore the relationship between frailty and sepsis-associated delirium (SAD). We classified patients using ICD-9 (severe sepsis: 995.62, septic shock: 785.52 [41]) and ICD-10 codes (septic shock: R65.2 [42]; severe sepsis with septic shock: R65.21 [43]). Additionally, participants were categorized into three groups based on the Modified Frailty Index (MFI): non-frail (MFI = 0), pre-frail (MFI = 1–2), and frail (MFI ≥ 3). This classification method particularly focused on pre-frailty stage patients, providing comprehensive information to assess the relationship between frailty and SAD.

## Results

### Baseline characteristics of the cohort

The study involved 11,740 older adults diagnosed with sepsis, with a median age of approximately 76 years (interquartile range [IQR]: 70.47–83.14 years). Detailed baseline characteristics are presented in Table 1, showing that 44.3% were female, and the majority (70.7%) were Caucasian. The median MFI score was 2 (IQR: 1–3). Patients were categorized into frail (*n* = 4,496) and non-frail (*n* = 7,244) groups based on their frailty status. Compared with non-frail patients, those with frailty had longer hospital and ICU stays (median hospital stay: 10.52 [IQR: 6.48–16.96] vs. 8.15 [IQR: 5.32–13.80] days, *p* < 0.001; median ICU stay: 3.93 [IQR: 2.16–7.73] vs. 3.02 [IQR: 1.79–5.70] days, *p* < 0.001). Additionally, they had worse hospital outcomes, including higher rates of SAD (55.7% vs. 44.6%) and in-hospital mortality (20.9% vs. 15.2%), with statistically significant differences (*p* < 0.05).

**Table 1:** Baseline patient characteristics in the cohorts

Variables	Overall (*n* = 11740)	Non-frail group (*n* = 7244)	Frail group (*n* = 4496)	*P*-value
Personal characteristics				
Age (years old)	76.36 (70.47, 83.14)	76.08 (70.11, 83.05)	76.76 (71.08, 83.22)	< 0.001
Sex (%)				< 0.001
Male	6540 (55.7)	3872 (53.5)	2668 (59.3)	
Female	5200 (44.3)	3372 (46.5)	1828 (40.7)	
Race (%)				< 0.001
White	8306 (70.7)	5235 (72.3)	3071 (68.3)	
Other	3434 (29.3)	2009 (27.7)	1425 (31.7)	
Hospital LOS (days)	8.93 (5.72, 15.04)	8.15 (5.32, 13.80)	10.52 (6.48, 16.96)	< 0.001
ICU LOS(days)	3.28 (1.92, 6.54)	3.02 (1.79, 5.70)	3.93 (2.16, 7.73)	< 0.001
MFI	2 (1, 3)	1 (1, 2)	3 (3, 4)	< 0.001
**Scores**				
GCS	13 (9, 14)	14 (10, 14)	13 (9, 14)	< 0.001
Braden score	14 (13, 16)	14 (13, 16)	14 (13, 16)	0.346
SOFA	6 (4, 9)	6 (4, 8)	6 (4, 9)	< 0.001
APSIII	51 (39, 69)	49 (37, 66)	55 (43, 72)	< 0.001
**Vital signs**				
Temperature, ℃	36.72 (36.39, 37.06)	36.67 (36.39, 37.00)	36.72 (36.44, 37.06)	< 0.001
Heart rate, beats/min	86 (74, 101)	86 (75, 101)	86 (74, 100)	0.061
MAP, mmHg	79 (69, 91)	79 (69, 91)	79 (69, 92)	0.289
Respiration rate, breaths/min	19 (16, 24)	19 (15, 23)	20 (16, 24)	< 0.001
**Laboratory parameters**				
White blood cell (10^9^/L)	11.3 (8.0, 15.9)	11.1 (7.8, 15.8)	11.5 (8.2, 16.0)	0.001
Red blood cell (10^9^/L)	3.41 (2.91, 3.96)	3.41 (2.91, 3.96)	3.41 (2.92, 3.96)	0.871
Hemoglobin (g/L)	10.1 (8.7, 11.8)	10.2 (8.7, 11.9)	10.0 (8.6, 11.7)	< 0.001
Platelet (10^9^/L)	185 (130.75, 256)	180 (127, 253)	192 (136, 262)	< 0.001
Albumin (g/dL)	3.3 (2.8, 3.8)	3.4 (2.8, 3.9)	3.3 (2.8, 3.8)	< 0.001
Lactate (mmol/L)	1.7 (1.2, 2.5)	1.70 (1.2, 2.5)	1.70 (1.2, 2.5)	0.463
**Comorbidities**				
Myocardial infarct (%)				< 0.001
Yes	2698 (23.0)	674 (9.3)	2024 (45.0)	
No	9042 (77.0)	6570 (90.7)	2472 (55.0)	
Congestive heart failure (%)				< 0.001
Yes	5052 (43.0)	2040 (28.2)	3012 (67.0)	
No	6688 (57.0)	5204 (71.8)	1484 (33.0)	
Chronic pulmonary disease (%)				< 0.001
Yes	3859 (32.9)	2013 (27.8)	1846 (41.1)	
No	7881 (67.1)	5231 (72.2)	2650 (58.9)	
Liver disease (%)				0.071
Yes	1210 (10.3)	776 (10.7)	434 (9.7)	
No	10,530 (89.7)	6468 (89.3)	4062 (90.3)	
Diabetes (%)				< 0.001
Yes	4277 (36.4)	1517 (20.9)	2760 (61.4)	
No	7463 (63.6)	5727 (79.1)	1736 (38.6)	
Renal disease (%)				< 0.001
Yes	3758 (32.0)	1710 (23.6)	2048 (45.6)	
No	7982 (68.0)	5534 (76.4)	2448 (54.4)	
Malignant cancer (%)				< 0.001
Yes	1826 (15.6)	1251 (17.3)	575 (12.8)	
No	9914 (84.4)	5993 (82.7)	3921 (87.2)	
Severe sepsis (%)				< 0.001
Yes	2928 (24.9)	1724 (23.8)	1204 (26.8)	
No	8812 (75.1)	5520 (76.2)	3292 (73.2)	
Depression (%)				0.004
Yes	1752 (14.9)	1026 (14.2)	726 (16.1)	
No	9988 (85.1)	6218 (85.8)	3770 (83.9)	
History of fall (%)				0.002
Yes	3186 (27.1)	1894 (26.1)	1292 (28.7)	
No	8554 (72.9)	5350 (73.9)	3204 (71.3)	
Cerebrovascular disease (%)				< 0.001
Yes	1842 (15.7)	722 (10.0)	1120 (24.9)	
No	9898 (84.3)	6522 (90.0)	3376 (75.1)	
Metastatic solid tumor (%)				< 0.001
Yes	772 (6.6)	581 (8.0)	191 (4.2)	
No	10,968 (93.4)	6663 (92.0)	4305 (95.8)	
Peptic ulcer disease (%)				1
Yes	406 (3.5)	251 (3.5)	155 (3.4)	
No	11,334 (96.5)	6993 (96.5)	4341 (96.6)	
Peripheral vascular disease (%)				< 0.001
Yes	1856 (15.8)	730 (10.1)	1126 (25.0)	
No	9884 (84.2)	6514 (89.9)	3370 (75.0)	
Paraplegia (%)				< 0.001
Yes	592 (5.0)	286 (3.9)	306 (6.8)	
No	11,148 (95.0)	6958 (96.1)	4190 (93.2)	
**Treatment and drugs**				
Invasive mechanical ventilation (%)				< 0.001
Yes	5779 (49.2)	3448 (47.6)	2331 (51.8)	
No	5961 (50.8)	3796 (52.4)	2165 (48.2)	
Sedatives (%)				0.044
Yes	8166 (69.6)	5088 (70.2)	3078 (68.5)	
No	3574 (30.4)	2156 (29.8)	1418 (31.5)	
Vasoactive agents (%)				0.917
Yes	6517 (55.5)	4018 (55.5)	2499 (55.6)	
No	5223 (44.5)	3226 (44.5)	1997 (44.4)	
Renal replacement therapy (%)				< 0.001
Yes	1201 (10.2)	543 (7.5)	658 (14.6)	
No	10,539 (89.8)	6701 (92.5)	3838 (85.4)	
**Outcomes**				
Delirium (%)				< 0.001
Yes	5732 (48.8)	3229 (44.6)	2503 (55.7)	
No	6008 (51.2)	4015 (55.4)	1993 (44.3)	
In-hospital mortality (%)				< 0.001
Expired	2039 (17.4)	1098 (15.2)	941 (20.9)	
Alive	9701 (82.6)	6146 (84.8)	3555 (79.1)	

Abbreviations: APSIII, Acute Physiology Score III; GCS, Glasgow Coma Scale; ICU, intensive care unit; LOS, length of stay; MAP, mean arterial blood pressure; MFI, modified frailty index; SOFA, sequential organ failure assessment

Frailty status was defined based on the modified frailty index (MFI) score: a score greater than 3 indicates frailty, a score of 1–2 indicates pre-frailty, and a score of 0 indicates no frailty. For the purposes of this study, pre-frailty was dichotomized. Patients categorized as “pre-frail” and “non-frail” are collectively referred to as “non-frail” patients

Continuous variables are presented as median (interquartile range), and categorical variables are presented as numbers (percentages)

P-values were calculated using the Wilcoxon rank sum test for continuous variables and the chi-squared test or Fisher’s exact test for categorical variables

### Association between frailty and SAD

Multivariable logistic regression analysis revealed a significant positive association between MFI scores and the risk of SAD in older adults (adjusted OR: 1.13, 95% CI: 1.09–1.17, *p* < 0.001) (Table 2). Further stratification by frailty status showed that frail older adults with multiple comorbidities had a significantly higher risk of developing SAD compared to non-frail counterparts (OR: 1.31, 95% CI: 1.20–1.43, *p* < 0.001, E-value: 1.55) (Table 3). These findings indicate that frailty is an independent risk factor for SAD development.

**Table 2:** Association between MFI score (continuous variable) and Sepsis-Associated Delirium in older adults

Regression analysis	OR	95% CI	*P*-value
Unadjusted model	1.21	1.18–1.24	< 0.001
Adjusted model	1.13	1.09–1.17	< 0.001
PSM model	1.16	1.13–1.19	< 0.001
IPTW model	1.14	1.10–1.17	< 0.001

Abbreviations: MFI, modified frailty index; CI, confidence interval; OR, odds ratio; PSM, Propensity Score-matched; IPTW, Inverse Probability of Treatment Weighting

Note: The multivariate model was adjusted for age, sex, race, weight, glasgow coma scale, acute physiology score III, sequential organ failure assessment, Braden score, platelet, red blood cell, white blood cell, albumin, temperature, mean blood pressure, heart rate, respiratory rate hemoglobin, lactate, cerebrovascular disease, history of fall, invasive mechanical ventilation, and use of sedatives; The PSM and IPTW models were adjusted for the same variables as the multivariate model

**Table 3:** Association between Frailty Status (categorical variable) and Sepsis-Associated Delirium in older adults

Regression analysis	OR	95% CI	*P*-value	E-value (lower limit of the 95% CI)
Unadjusted model	1.56	1.45–1.68	< 0.001	1.81 (1.7)
Adjusted model	1.31	1.20–1.43	< 0.001	1.55 (1.42)
PSM model	1.35	1.24–1.47	< 0.001	1.6 (1.47)
IPTW model	1.26	1.16–1.37	< 0.001	1.49 (1.37)

Abbreviations: CI, confidence interval; OR, odds ratio; PSM, Propensity Score-matched; IPTW, Inverse Probability of Treatment Weighting

Note: The multivariate model was adjusted for age, sex, race, weight, glasgow coma scale, acute physiology score III, sequential organ failure assessment, Braden score, platelet, red blood cell, white blood cell, albumin, temperature, mean blood pressure, heart rate, respiratory rate hemoglobin, lactate, cerebrovascular disease, history of fall, invasive mechanical ventilation, and use of sedatives; The PSM and IPTW models were adjusted for the same variables as the multivariate model. The reference group consists of non-frail patients. Frailty status was defined based on the modified frailty index (MFI) score: a score greater than 3 indicates frailty, a score of 1–2 indicates pre-frailty, and a score of 0 indicates no frailty. For the purposes of this study, pre-frailty was dichotomized. Patients categorized as “pre-frail” and “non-frail” are collectively referred to as “non-frail” patients

### Patient survival in different risk groups

**Fig. 2:**
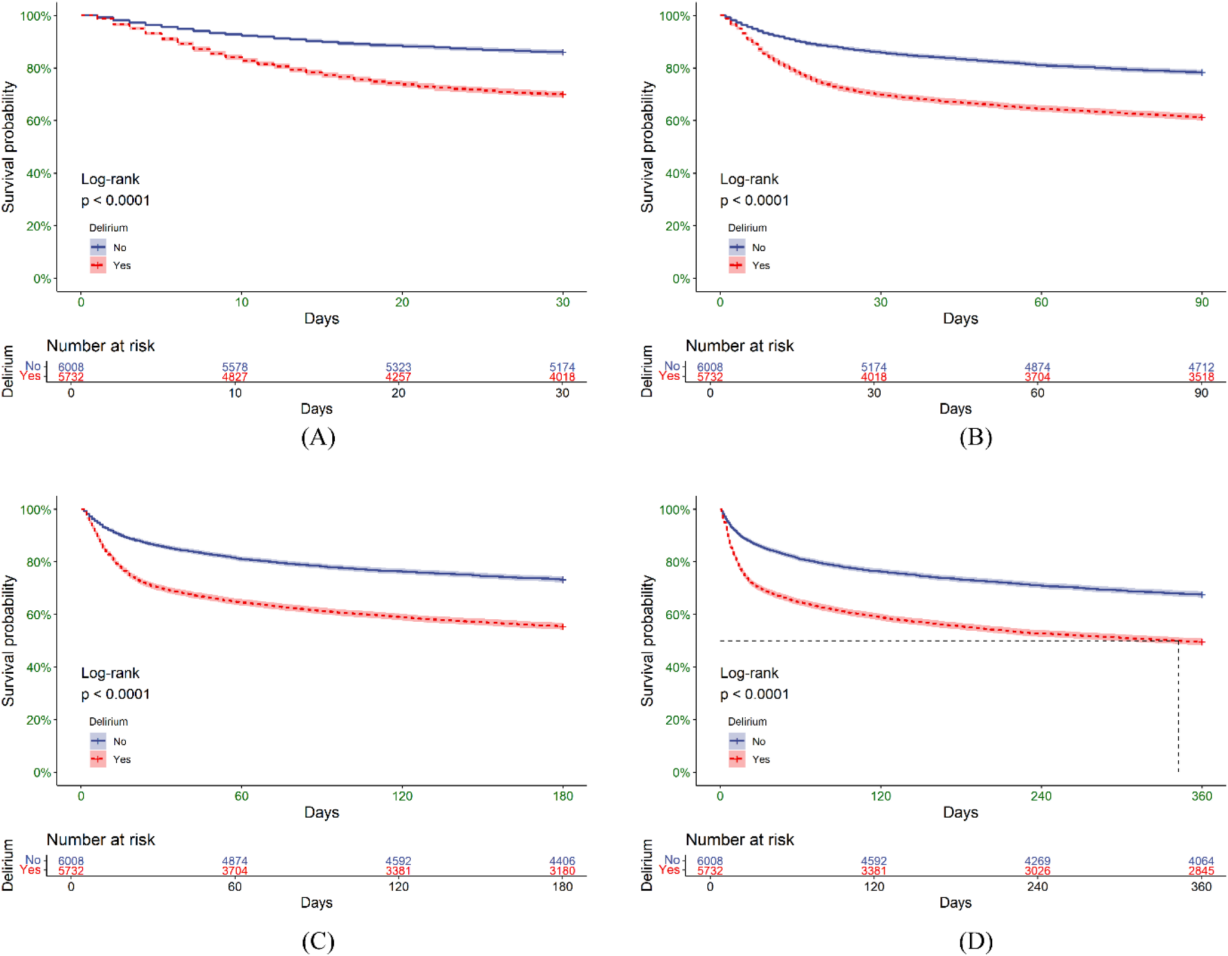
Kaplan-Meier survival curves for sepsis in older adults with and without delirium. Note: This figure shows the Kaplan-Meier survival curves comparing the survival probabilities of older adults with sepsis with and without delirium over different time periods. The curves show the probability of survival over 30 days (**A**), 90 days (**B**), 180 days (**C**), and 360 days (**D**). The log-rank test was used to compare the survival distributions between the two groups, and p-values indicate significant differences

The Kaplan-Meier survival curves (Fig. 2) demonstrate significant differences in survival rates between older adults with sepsis with and without delirium (Log-rank test; *P* < 0.001). At 30 days (Fig. 2A), 90 days (Fig. 2B), 180 days (Fig. 2C), and 360 days (Fig. 2D), the delirium group consistently showed significantly lower survival probabilities compared to the non-delirium group (*p* < 0.001). These findings indicate that delirium significantly impacts both short-term and one-year survival in critically ill older adults with sepsis.

### Subgroup analysis

**Fig. 3:**
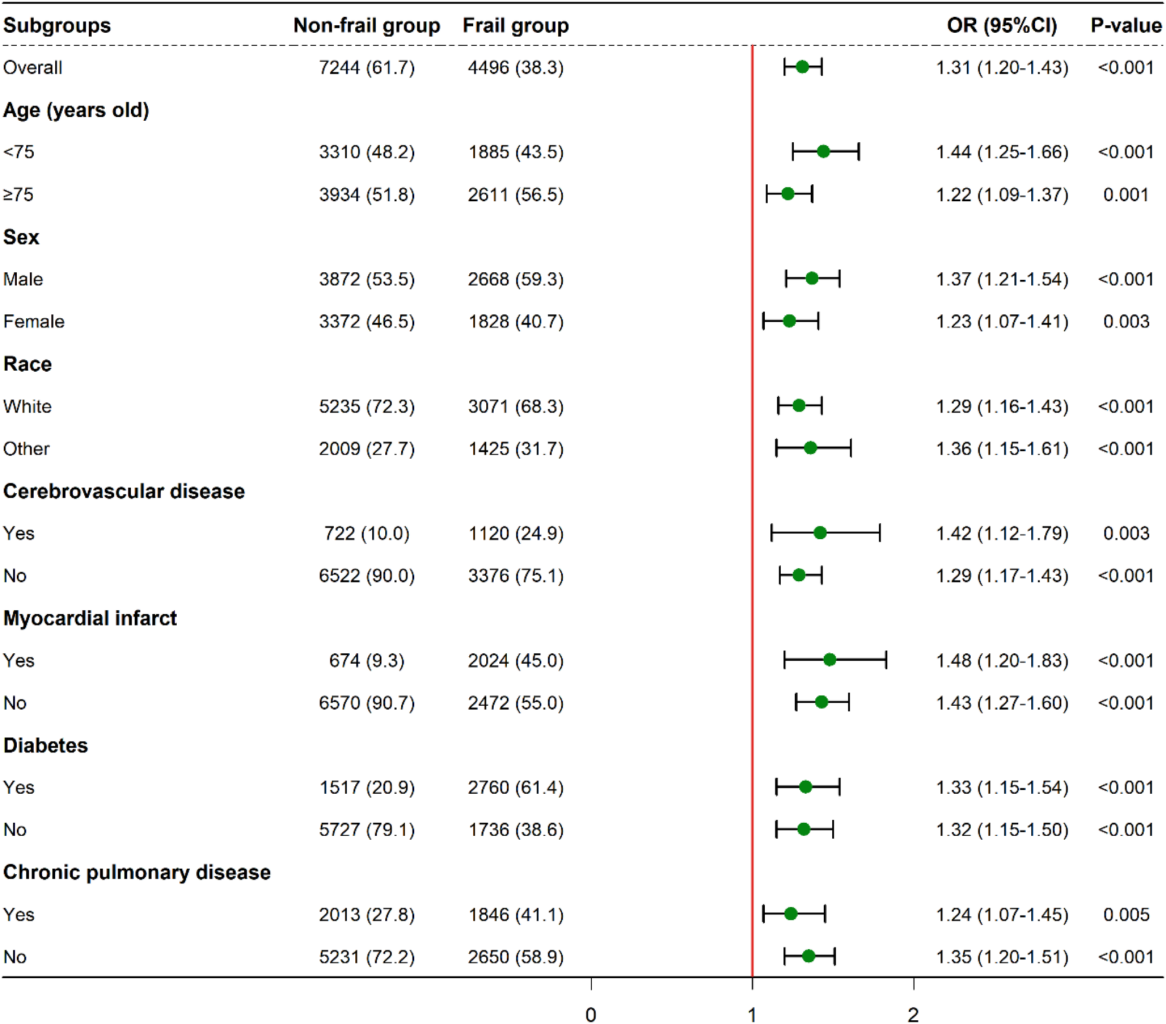
Subgroup Analysis of Delirium Risk by Frailty Status in Older Adults. Note: This figure shows a subgroup analysis comparing the risk of delirium between non-frail and frail groups of critically ill older adults. Odds ratios (OR) with 95% confidence intervals (CI) and p-values are shown for several demographic and clinical characteristics. Abbreviations: OR, Odds Ratio; CI, Confidence Interval

Subgroup analysis was conducted to investigate the association between frailty and the risk of SAD across different subpopulations (Fig. 3). Significant differences were observed across all seven subgroups analyzed, which included age, sex, race, history of cerebrovascular disease, myocardial infarction, diabetes, and chronic lung disease. These findings suggest that the MFI is linked to an elevated risk of SAD across diverse demographic and clinical subgroups.

### Sensitivity analysis

In this study, propensity score matching (PSM) and inverse probability treatment weighting (IPTW) techniques were applied to the original cohort. The corresponding changes in standardized mean differences (SMD) are presented in Supplementary Fig. 2 and baseline characteristics after PSM are shown in Supplementary Table 4. Following adjustment, the majority of covariates exhibited SMDs below 0.1, suggesting enhanced balance between the groups. Results from the matched or weighted populations aligned with those of the original cohort, indicating that frailty, as assessed by the MFI, correlates with an elevated risk of SAD in older adult critically ill patients (Table 3).

The study explored the correlation between frailty and SAD in two particular subgroups of critically ill older adult patients. The findings indicate a significantly increased risk of delirium among hospitalized survivors and frail patients with non-severe sepsis or septic shock (*p* < 0.05) (Supplementary Table 2). Additionally, consistent results were observed when the cohort was divided into non-frail, pre-frail, and frail groups (Supplementary Table 3). Compared to the non-frail group, older adults in the pre-frail stage also demonstrated a higher likelihood of developing SAD (OR: 1.79, 95% CI: 1.31–2.45, *p* < 0.001).

The adjusted model’s e-value was 1.55 (95% CI lower limit: 1.42), suggesting that an unmeasured confounder would need to be associated with both frailty (exposure) and SAD (outcome) with a risk ratio of at least 1.55, beyond the influence of measured covariates, to fully explain or eliminate the observed association. Considering statistical uncertainty, the lower limit of 1.42 indicates that even under conservative estimation, a confounder of considerable strength would be required to render the observed association statistically non-significant.

## Discussion

Considering the potential adverse impact of delirium on septic patients, it is essential to thoroughly evaluate the risk of SAD development. Our study comprehensively examined septic patients using a large, open dataset. The results indicate that frailty quantified by the MFI independently predicts the risk of delirium, even after accounting for other potential influencing factors. Frail patients exhibited a significantly higher risk of developing SAD compared to non-frail patients. This indicates that frailty independently increases the risk of delirium in association with sepsis.

Frailty is emerging as one of the most significant global public health challenges of the next century [44, 45]. As the aging population rapidly expands, the number of frail older people is increasing, placing a significant burden on global healthcare systems [46]. With the global demographic shift toward an older population, frailty is becoming a critical area of research in clinical geriatrics, gerontology, and public health [47]. Studies have shown that frailty is not just a natural consequence of aging, but a major factor influencing quality of life and health outcomes in older adults [48]. As a prevalent geriatric syndrome, frailty is strongly associated with adverse outcomes such as falls [49], delirium [50], hospitalization [51], disability [52], and death [53]. Cheng and colleagues demonstrated that frailty independently predicted postoperative delirium in older cardiac surgery patients using the MFI [31]. Our study extends this finding to sepsis patients, providing new evidence that the predictive role of frailty for delirium persists in a distinct and vulnerable population of critically ill older adults. Previous research has shown that frailty scores are independent predictors of delirium, with frailty showing strong predictive ability for delirium [54]. An updated meta-analysis found that frail patients have a 2.96-fold increased risk of delirium, highlighting the need for early frailty screening and comprehensive delirium prevention [55]. While previous studies were limited by small sample sizes, our analysis of 11,740 older sepsis patients provides robust evidence supporting the frailty-delirium association. Our findings confirm that frailty independently predicts SAD after adjusting for potential confounders. The significantly higher SAD risk in frail, multimorbid patients compared to their non-frail counterparts emphasizes frailty’s crucial role in delirium development. This study underscores the importance of addressing frailty in the clinical management of older adult sepsis patients to reduce the risk of delirium.

Frailty may predispose individuals to an increased risk of SAD, though the underlying pathophysiological pathways remain incompletely understood and warrant comprehensive investigation. Sepsis is recognized as a systemic inflammatory response syndrome triggered by infection, resulting in an excessive immune activation that causes extensive tissue damage and multiple organ failure [2, 3]. In frail individuals, characterized by diminished physiological reserves from multifactorial causes, there is an associated decline in immune and anti-inflammatory capabilities. Such impairments may exacerbate the inflammatory response in sepsis, thus heightening the risk of developing SAD [47, 56]. Moreover, the severe inflammation in sepsis extends beyond the peripheral system, compromising the blood-brain barrier’s integrity. This disruption facilitates the infiltration of inflammatory mediators and toxins into the central nervous system (CNS), directly harming neurons and neural circuits [7, 55]. Frailty, with its associated weakened physiological barriers and repair mechanisms, likely reduces the capacity to prevent these invasions, thereby increasing CNS vulnerability [55]. Disturbances in neurotransmitter systems, influenced by both sepsis and frailty, can disrupt communication within the brain and between the brain and other organs, potentially contributing to the manifestation of SAD symptoms. These disturbances notably impact mood regulation, attention, memory, and cognitive functions [57, 58]. In conclusion, frailty increases the risk of SAD through multiple mechanisms, including exacerbation of inflammatory responses, damage to the blood-brain barrier, disruption of neurotransmitter balance, and effects on energy-nutrient metabolism. Future research should aim to delineate the specific pathways and interrelationships of these mechanisms in order to provide more precise and effective strategies for the prevention and treatment of SAD.

Our findings have important clinical implications, particularly for ICU sepsis patients with pre-existing frailty. The robust association between frailty and SAD highlights the need to assess and manage frailty in the ICU setting. Frailty may increase the risk of SAD through several mechanisms, including neurotransmitter dysregulation and immune system dysfunction [47, 55, 56]. Therefore, ICU staff should closely monitor patients’ frailty status and take proactive measures to prevent delirium in ICU patients with sepsis. Specifically, standardized frailty assessment tools such as the MFI or the Fried frailty phenotype [10] should be used for new ICU sepsis admissions to rapidly identify high-risk patients. Individualized care plans should be developed based on these assessments. These plans may include enhanced nutritional support to improve nutritional status, designed exercise programs to gradually restore muscle strength and function, and necessary psychological support to alleviate anxiety and depression, thereby comprehensively improving the patient’s overall health [48, 59].

Clinical practice for frail older adults requires a targeted approach that addresses their unique needs and complexities, beginning with early frailty screening and precise case identification [47]. A multidisciplinary, collaborative management strategy that integrates nutrition, exercise, pharmacology, and cognitive rehabilitation should be used to provide comprehensive care. This includes optimizing nutritional plans to meet physical needs, promoting activities of daily living to maintain physical function, careful medication management to reduce adverse effects, and implementing cognitive training to keep the brain active [60, 61]. In addition, a number of delirium prevention measures should be actively implemented in the ICU setting. These measures include optimizing the unit environment by reducing noise and light disturbances to create a more comfortable resting environment, establishing and maintaining regular schedules to help patients maintain stable circadian rhythms, providing individualized cognitive stimulation activities to promote recovery of brain function, and strictly managing the use of sedatives to avoid unnecessary drug-induced delirium [62, 63]. Through the systematic implementation of these targeted preventive measures, with particular emphasis on frail elderly patients, the incidence of SAD can be effectively reduced, leading to enhanced recovery from sepsis and improved patient outcomes in terms of both quality of life and prognosis.

### Limitations

This study has several notable limitations. A primary methodological limitation concerns the frailty assessment tool used, the MFI. Although the MFI has been validated in previous studies and offers practical advantages in the ICU setting, it reflects only a predefined set of 11 comorbidities and may not capture all dimensions of frailty, particularly cognitive function, which is critical in the assessment of delirium. As a single-center retrospective observational study, its scope is relatively limited, potentially affecting the generalizability and external validity of the research findings. Although a significant association between frailty and SAD was observed, the study design does not establish a clear causal relationship. Due to limitations in the database, key variables such as activities of daily living were not adequately captured. Furthermore, the use of both ICD-9 and ICD-10 codes in the MIMIC-IV database may lead to inaccuracies in the calculation of the MFI, and the assessment of patient functional status using ICD-9 code R26 may introduce subjectivity and inherent limitations. The study also did not consider specific details such as the type, duration, and severity of delirium, which may limit our understanding of the complex relationship between frailty and delirium.

Future multicenter prospective studies with larger sample sizes would enhance the generalizability and reliability of findings. Such studies should consider employing more comprehensive frailty assessment tools, such as Fried’s Frailty Phenotype [10] or Rockwood’s Frailty Index [64], which provide a more holistic evaluation by incorporating multiple domains, including cognitive function, physical performance, and functional status. Additionally, a more detailed characterization of delirium features would provide deeper insights into the relationship between frailty and delirium. These advancements would contribute to a better understanding of the causal relationship between frailty and delirium, ultimately informing evidence-based prevention and treatment strategies for sepsis-associated delirium in frail older adults.

## Conclusions

This study demonstrated the association between frailty and SAD in ICU sepsis patients. These findings have significant clinical relevance for the prediction and prevention of SAD in these patients. Additionally, our study highlights the association between SAD in older adult patients and adverse short-term and one-year clinical outcomes, emphasizing the importance of delirium prevention. Healthcare professionals are advised to prioritize early identification and intervention of frailty and SAD in clinical practice to improve patient outcomes.

## Electronic supplementary material

Below is the link to the electronic supplementary material.

Supplementary Material 1

## Data Availability

The data were available on the MIMIC-IV website at https://mimic.physionet.org/. The data in this article can be reasonably applied to the corresponding author.
