# Supplementary material for: Frailty as an independent risk factor for sepsis-associated delirium: a cohort study of 11,740 older adult ICU patients
Source: Aging Clin Exp Res. 2025 Feb 27;37(1):52. doi: 10.1007/s40520-025-02956-2 (PMC11865144; doi:10.1007/s40520-025-02956-2)
Supplement: Supplementary file 1 — Supplementary Material 1 [file 40520_2025_2956_MOESM1_ESM.docx]

**Supplementary Table 1** ICD-9 and ICD-10 codes in modified frailty index development

| **No.** | **Items** | **ICD-10 Description** | **ICD-10 Code** | **ICD-9 Code** |
| --- | --- | --- | --- | --- |
| **1** | History of Diabetes Mellitus | Diabetes mellitus due to underlying condition; Drug or chemical induced diabetes mellitus; Type 1 diabetes mellitus; Type 2 diabetes mellitus; Other specified diabetes mellitus; Encounter for screening for diabetes mellitus. | E08* OR E09* OR E10* OR E11* OR E13* OR Z131* | 249* OR 250* OR 3572 OR V771 |
| **2** | History of Congestive Heart Failure | Heart failure, unspecified. | I50.9 | 4280 |
| **3** | History of Hypertension Requiring Medication | Hypertensive disease. | I1* | 40* |
| **4** | History of either Transient Ischemic Attack or Cerebrovascular Accident | Transient cerebral ischemic attacks and related syndromes; Nontraumatic subarachnoid hemorrhage; Nontraumatic intracerebral hemorrhage; Other and unspecified nontraumatic intracranial hemorrhage; Cerebral infarction; Personal history of transient ischemic attack (TIA), and cerebral infarction without residual deficits. | G45* OR I60* OR I61* OR I62* OR I63* OR Z86.73 | 430 OR 431 OR 432* OR 43301 OR 43311 OR 43321 OR 43331 OR 43381 OR 43391 OR 43401 OR 43411 OR 43491 OR 435* OR V1254 |
| **5** | Functional Status (Loss Function) | Abnormalities of gait and mobility. | R26* | 7197 OR 7812 |
| **6** | History of Myocardial Infarction | ST elevation (STEMI) and non-ST elevation (NSTEMI) myocardial infarction; Subsequent ST elevation (STEMI) and non-ST elevation (NSTEMI) myocardial infarction; Certain current complications following ST elevation (STEMI) and non-ST elevation (NSTEMI) myocardial infarction (within the 28 days period); Old myocardial infarction; Cardiac septal defect, acquired. | I21* OR I22* OR I23* OR I252 OR I510 | 410* OR 4110 OR 412 OR 4297* |
| **7** | History of either Peripheral Vascular Disease or Rest Pain | Atherosclerosis of renal artery; Atherosclerosis of native arteries of the extremities; Atherosclerosis of other arteries; Other and unspecified atherosclerosis; Other aneurysm; Other peripheral vascular diseases; Embolism and thrombosis of arteries of the upper extremities; Embolism and thrombosis of arteries of the lower extremities; Embolism and thrombosis of arteries of extremities, unspecified; Embolism and thrombosis of iliac artery; Embolism and thrombosis of other arteries; Embolism and thrombosis of unspecified artery; Atheroembolism; Phlebitis and thrombophlebitis; Portal vein thrombosis; Other venous embolism and thrombosis; Varicose veins of lower extremities; Esophageal varices; Varicose veins of other sites; Personal history of thrombophlebitis. | I70.1 OR I70.2* OR I70.8 OR I70.9* OR I72* OR I73* OR I74.2 OR I74.3 OR I74.4 OR I74.5 OR I74.8 OR I74.9 OR I75* OR I80* OR I81* OR I82* OR I83* OR I85* OR I86* OR Z8672 | 4401 OR 4402* OR 4404 OR 4408 OR 4409 OR 442* OR 443* OR 4442* OR 4448* OR 4449 OR 445* OR 451* OR 452* OR 453* OR 454* OR 456* OR V1252 |
| **8** | History of Cerebrovascular Accident with Neurological Deficit | Sequelae of cerebrovascular disease. | I69* | 438* |
| **9** | History of either COPD or Pneumonia | Viral pneumonia, not elsewhere classified; Pneumonia due to Streptococcus pneumoniae; Pneumonia due to Hemophilus influenzae; Bacterial pneumonia, not elsewhere classified; Pneumonia due to other infectious organisms, not elsewhere classified; Pneumonia in diseases classified elsewhere; Pneumonia, unspecified organism; Emphysema; Other chronic obstructive pulmonary disease; Personal history of pneumonia (recurrent). | J12* OR J13* OR J14* OR J15* OR J16* OR J17* OR J18* OR J43* OR J44* OR Z78.01 | 480* OR 481* OR 482* OR 483* OR 484* OR 485* OR 486* OR 491* OR 492* OR V1261 |
| **10** | History of either prior PCI, CABG, or angina | Angina pectoris; Atherosclerotic heart disease of native coronary artery with angina pectoris; Atherosclerosis of coronary artery bypass graft(s), unspecified, with angina pectoris; Atherosclerosis of autologous vein coronary artery bypass graft(s) with angina pectoris; Atherosclerosis of autologous artery coronary artery bypass graft(s) with angina pectoris; Atherosclerosis of non-autologous biological coronary artery bypass graft(s) with angina pectoris; Atherosclerosis of other coronary artery bypass graft(s) with angina pectoris; Presence of aortocoronary bypass graft; Presence of coronary angioplasty implant and graft; Coronary angioplasty status. | I20* OR I2511 OR I2570 OR I2571 OR I2572 OR I2573 OR I2579 OR Z951 OR Z955 OR Z9861 | 4111 OR 413* OR 41402 OR 41403 OR 41404 OR 41405 OR V4581 OR V4582 OR V472 |
| **11** | History of Impaired Sensorium | Disturbances of skin sensation; Disturbances of smell and taste; Visual disturbances; Blindness and low vision; Conductive and sensorineural hearing loss; Other and unspecified hearing loss; Dual sensory impairment. | R20* OR R43* OR H53* OR H54* OR H90* OR H91* OR Z73.82 | 7820 OR 7811 OR V415 OR 368* OR 369* OR V410 OR 389* OR 3881* OR 3882 OR V412 OR V4985 OR V484 OR V485 OR V493 |

Abbreviations: ICD: International Classification of Diseases.

Note:

"*" means all subcodes contained in this code.

"AND", "OR" and "NOT" follow the logic of Boolean operations.

References:

Hao B, Chen T, Qin J, Meng W, Bai W, Zhao L, Ou X, Liu H, Xu W. A comparison of three approaches to measuring frailty to determine adverse health outcomes in critically ill patients. Age Ageing. 2023 Jun 1;52(6):afad096. doi: 10.1093/ageing/afad096;

Cheng H, Ling Y, Li Q, Li X, Tang Y, Guo J, Li J, Wang Z, Ming WK, Lyu J. Association between modified frailty index and postoperative delirium in patients after cardiac surgery: A cohort study of 2080 older adults. CNS Neurosci Ther. 2024 Jun;30(6):e14762. doi: 10.1111/cns.14762.

**Supplementary Table 2** Baseline patient characteristics after propensity score matching.

| **Variables** | **Overall (n=8992)** | **Non-frail group (n=4496)** | **Frail group (n=4496)** | **P-value** |
| --- | --- | --- | --- | --- |
| **Personal characteristics** |  |  |  |  |
| Age (years old) | 76.81 (70.82, 83.57) | 76.91 (70.60, 83.95) | 76.76 (71.08, 83.22) | 0.945 |
| Sex (%) |  |  |  | 0.668 |
| Male | 5315 (59.1) | 2647 (58.9) | 2668 (59.3) |  |
| Female | 3677 (40.9) | 1849 (41.1) | 1828 (40.7) |  |
| Race (%) |  |  |  | 0.285 |
| White | 6190 (68.8) | 3119 (69.4) | 3071 (68.3) |  |
| Other | 2802 (31.2) | 1377 (30.6) | 1425 (31.7) |  |
| Hospital LOS (days) | 9.55 (5.92, 15.91) | 8.63 (5.48, 14.78) | 10.52 (6.48, 16.96) | <0.001 |
| ICU LOS(days) | 3.57 (2.02, 7.06) | 3.26 (1.91, 6.36) | 3.93 (2.16, 7.73) | <0.001 |
| MFI | 2.5 (1, 3) | 1 (1, 2) | 3 (3, 4) | <0.001 |
| **Scores** |  |  |  |  |
| GCS | 13 (9, 14) | 13 (9, 14) | 13 (9, 14) | <0.001 |
| Braden score | 14 (13, 16) | 14 (13, 16) | 14 (1, 16) | 0.083 |
| SOFA | 6 (4, 9) | 6 (4, 9) | 6 (4, 9) | 0.029 |
| APSIII | 54 (42, 72) | 53 (41, 71.25) | 55 (43, 72) | 0.003 |
| **Vital signs** |  |  |  |  |
| Temperature, ℃ | 36.72 (36.44, 37.06) | 36.72 (36.44, 37.06) | 36.72 (36.44, 37.06) | 0.736 |
| Heart rate, beats/min | 86 (74, 100) | 85 (74, 100) | 86 (74, 100) | 0.79 |
| MBP, mmHg | 79 (68, 92) | 79 (68, 92) | 79 (69, 92) | 0.228 |
| Respiration rate, breaths/min | 20 (16, 24) | 19.5 (16, 24) | 20 (16, 24) | 0.071 |
| **Laboratory parameters** |  |  |  |  |
| White blood cell (10^9^/L) | 11.4 (8.0, 16.0) | 11.3 (7.8, 16.1) | 11.5 (8.2, 16.0) | 0.072 |
| Red blood cell (10^9^/L) | 3.41 (2.90, 3.96) | 3.42 (2.89, 3.96) | 3.41 (2.92, 3.96) | 0.693 |
| Hemoglobin (g/L) | 10.0 (8.6, 11.7) | 10.1 (8.6, 11.7) | 10.0 (8.6, 11.7) | 0.503 |
| Platelet (10^9^/L) | 188 (132.75, 260) | 185 (129, 259) | 192 (136, 262) | 0.005 |
| Albumin (g/dL) | 3.3 (2.8, 3.8) | 3.3 (2.8, 3.8) | 3.3 (2.8, 3.8) | 0.95 |
| Lactate (mmol/L) | 1.7 (1.2, 2.5) | 1.7 (1.2, 2.5) | 1.7 (1.2, 2.5^1^) | 0.909 |
| **Comorbidities** |  |  |  |  |
| Myocardial infarct (%) |  |  |  | <0.001 |
| Yes | 2435 (27.1) | 411 (9.1) | 2024 (45.0) |  |
| No | 6557 (72.9) | 4085 (90.9) | 2472 (55.0) |  |
| Congestive heart failure (%) |  |  |  | <0.001 |
| Yes | 4391 (48.8) | 1379 (30.7) | 3012 (67.0) |  |
| No | 4601 (51.2) | 3117 (69.3) | 1484 (33.0) |  |
| Chronic pulmonary disease (%) |  |  |  | <0.001 |
| Yes | 3069 (34.1) | 1223 (27.2) | 1846 (41.1) |  |
| No | 5923 (65.9) | 3273 (72.8) | 2650 (58.9) |  |
| Liver disease (%) |  |  |  | 0.001 |
| Yes | 964 (10.7) | 530 (11.8) | 434 (9.7) |  |
| No | 8028 (89.3) | 3966 (88.2) | 4062 (90.3) |  |
| Diabetes (%) |  |  |  | <0.001 |
| Yes | 3773 (42.0) | 1013 (22.5) | 2760 (61.4) |  |
| No | 5219 (58.0) | 3483 (77.5) | 1736 (38.6) |  |
| Renal disease (%) |  |  |  | <0.001 |
| Yes | 3319 (36.9) | 1271 (28.3) | 2048 (45.6) |  |
| No | 5673 (63.1) | 3225 (71.7) | 2448 (54.4) |  |
| Malignant cancer (%) |  |  |  | <0.001 |
| Yes | 1381 (15.4) | 806 (17.9) | 575 (12.8) |  |
| No | 7611 (84.6) | 3690 (82.1) | 3921 (87.2) |  |
| Severe sepsis (%) |  |  |  | 0.343 |
| Yes | 2449 (27.2) | 1245 (27.7) | 1204 (26.8) |  |
| No | 6543 (72.8) | 3251 (72.3) | 3292 (73.2) |  |
| Depression (%) |  |  |  | 0.003 |
| Yes | 1350 (15.0) | 624 (13.9) | 726 (16.1) |  |
| No | 7642 (85.0) | 3872 (86.1) | 3770 (83.9) |  |
| History of fall (%) |  |  |  | 0.852 |
| Yes | 2575 (28.6) | 1283 (28.5) | 1292 (28.7) |  |
| No | 6417 (71.4) | 3213 (71.5) | 3204 (71.3) |  |
| Cerebrovascular disease (%) |  |  |  | <0.001 |
| Yes | 1835 (20.4) | 715 (15.9) | 1120 (24.9) |  |
| No | 7157 (79.6) | 3781 (84.1) | 3376 (75.1) |  |
| Metastatic solid tumor (%) |  |  |  | <0.001 |
| Yes | 562 (6.2) | 371 (8.3) | 191 (4.2) |  |
| No | 8430 (93.8) | 4125 (91.7) | 4305 (95.8) |  |
| Peptic ulcer disease (%) |  |  |  | 0.339 |
| Yes | 328 (3.6) | 173 (3.8) | 155 (3.4) |  |
| No | 8664 (96.4) | 4323 (96.2) | 4341 (96.6) |  |
| Peripheral vascular disease (%) |  |  |  | <0.001 |
| Yes | 1608 (17.9) | 482 (10.7) | 1126 (25.0) |  |
| No | 7384 (82.1) | 4014 (89.3) | 3370 (75.0) |  |
| Paraplegia (%) |  |  |  | <0.001 |
| Yes | 530 (5.9) | 224 (5.0) | 306 (6.8) |  |
| No | 8462 (94.1) | 4272 (95.0) | 4190 (93.2) |  |
| **Treatment and drugs** |  |  |  |  |
| Invasive mechanical ventilation (%) |  |  |  | 0.055 |
| Yes | 4570 (50.8) | 2239 (49.8) | 2331 (51.8) |  |
| No | 4422 (49.2) | 2257 (50.2) | 2165 (48.2) |  |
| Sedatives (%) |  |  |  | 0.288 |
| Yes | 6108 (67.9) | 3030 (67.4) | 3078 (68.5) |  |
| No | 2884 (32.1) | 1466 (32.6) | 1418 (31.5) |  |
| Vasoactive agents (%) |  |  |  | 0.106 |
| Yes | 5075 (56.4) | 2576 (57.3) | 2499 (55.6) |  |
| No | 3917 (43.6) | 1920 (42.7) | 1997 (44.4) |  |
| Renal replacement therapy (%) |  |  |  | <0.001 |
| Yes | 1100 (12.2) | 442 (9.8) | 658 (14.6) |  |
| No | 7892 (87.8) | 4054 (90.2) | 3838 (85.4) |  |
| **Outcomes** |  |  |  |  |
| Delirium (%) |  |  |  | <0.001 |
| Yes | 4669 (51.9) | 2166 (48.2) | 2503 (55.7) |  |
| No | 4323 (48.1) | 2330 (51.8) | 1993 (44.3) |  |
| In-hospital mortality (%) |  |  |  | <0.001 |
| Alive | 7249 (80.6) | 3694 (82.2) | 3555 (79.1) |  |
| Expired | 1743 (19.4) | 802 (17.8) | 941 (20.9) |  |

Abbreviations: APSIII, Acute Physiology Score III; GCS, Glasgow Coma Scale; ICU, intensive care unit; LOS, length of stay; MAP, mean arterial blood pressure; MFI, modified frailty index; SOFA, sequential organ failure assessment.

Note:

Frailty status was defined based on the modified frailty index (MFI) score: a score greater than 3 indicates frailty, a score of 1-2 indicates pre-frailty, and a score of 0 indicates no frailty. For the purposes of this study, pre-frailty was dichotomized. Patients categorized as "pre-frail" and "non-frail" are collectively referred to as "non-frail" patients.

Continuous variables are presented as median (interquartile range), and categorical variables are presented as numbers (percentages).

P-values were calculated using the Wilcoxon rank sum test for continuous variables and the chi-squared test or Fisher's exact test for categorical variables.

**Supplementary Table 3** Sensitivity analysis: Association between frailty and sepsis-associated delirium in special populations.

|  | Non-frail group^#^ | Frail group | P-value |
| --- | --- | --- | --- |
|  |  | OR (95% CI) |  |
| Model 1^$^  Unadjusted  Adjusted | Reference  Reference | 1.52 (1.40, 1.66)  1.27 (1.15, 1.40) | <0.001*  <0.001* |
| Model 2^$^  Unadjusted  Adjusted | Reference  Reference | 1.60 (1.47, 1.74)  1.33 (1.19, 1.47) | <0.001*  <0.001* |

Abbreviations: OR: odds ratios; CI: confidence intervals.

Note:

^$^Model 1 excluded in-hospital deaths and analyzed only hospital survivors (n=9701). Model 2 excluded patients with severe sepsis or septic shock and analyzed non-severe sepsis patients (n=8812).

^#^In this study, frailty status was assessed using the Modified Frailty Index (MFI) and categorized into a binary classification. An MFI score greater than 3 defined frailty, 1-2 indicated pre-frailty, and 0 indicated no frailty. For the purpose of this research, pre-frail and non-frail patients were collectively classified as "non-frail," resulting in two categories: frail and non-frail.

**Supplementary Table 4** Association Frailty (Three Categories) and Sepsis-Associated Delirium.

|  | Non-frail group | Pre-frail group | P-value | Frail group | P-value |
| --- | --- | --- | --- | --- | --- |
|  |  | OR (95% CI) |  | OR (95% CI) |  |
| Unadjusted | Reference | 1.85 (1.43, 2.40) | <0.001 | 2.34 (1.79, 3.05) | <0.001 |
| Adjusted | Reference | 1.79 (1.31, 2.45) | <0.001 | 1.89 (1.37, 2.63) | <0.001 |

Abbreviations: CI, confidence interval; OR, odds ratio; PSM, Propensity Score-matched; IPTW, Inverse Probability of Treatment Weighting.

Note: The multivariate model was adjusted for age, sex, race, weight, glasgow coma scale, acute physiology score III, sequential organ failure assessment, Braden score, platelet, red blood cell, white blood cell, albumin, temperature, mean blood pressure, heart rate, respiratory rate hemoglobin, lactate, cerebrovascular disease, history of fall, invasive mechanical ventilation, and use of sedatives.

Frailty status was defined based on the modified frailty index (MFI) score: a score greater than 3 indicates frailty, a score of 1-2 indicates pre-frailty, and a score of 0 indicates no frailty.


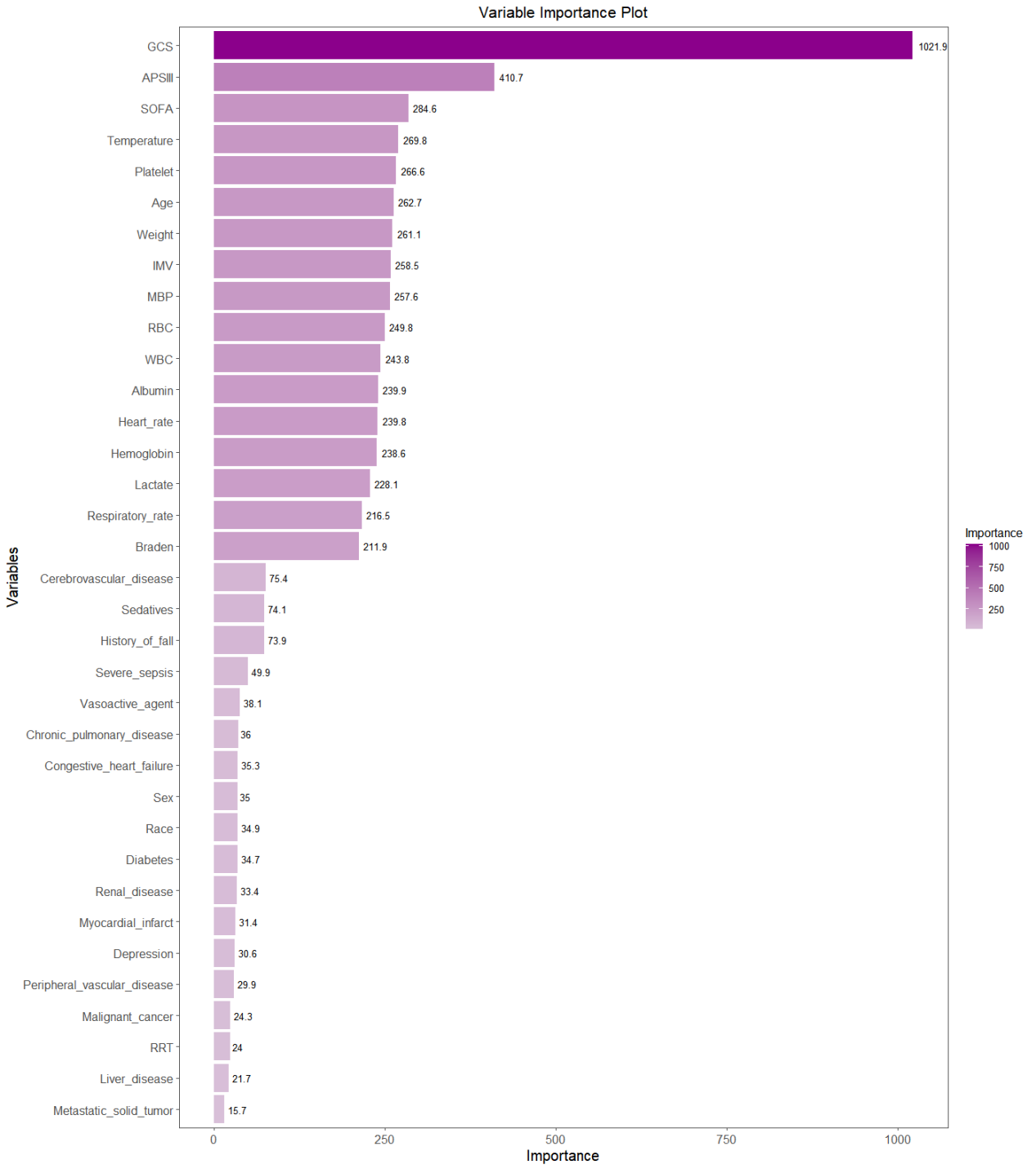


**Supplementary Figure 1** Variable importance plot for predicting sepsis-associated delirium using random forest method

Abbreviations: GCS, Glasgow Coma Scale; APSIII, Acute Physiology Score III; SOFA, Sequential Organ Failure Assessment; IMV, Invasive Mechanical Ventilation; MBP, Mean Blood Pressure; RBC, Red Blood Cell count; WBC, White Blood Cell count; RRT, Renal Replacement Therapy.

Note: This figure displays the relative importance of various clinical variables in predicting sepsis-associated delirium in critically ill patients. The variables are ranked based on their importance scores, with higher scores indicating greater importance.


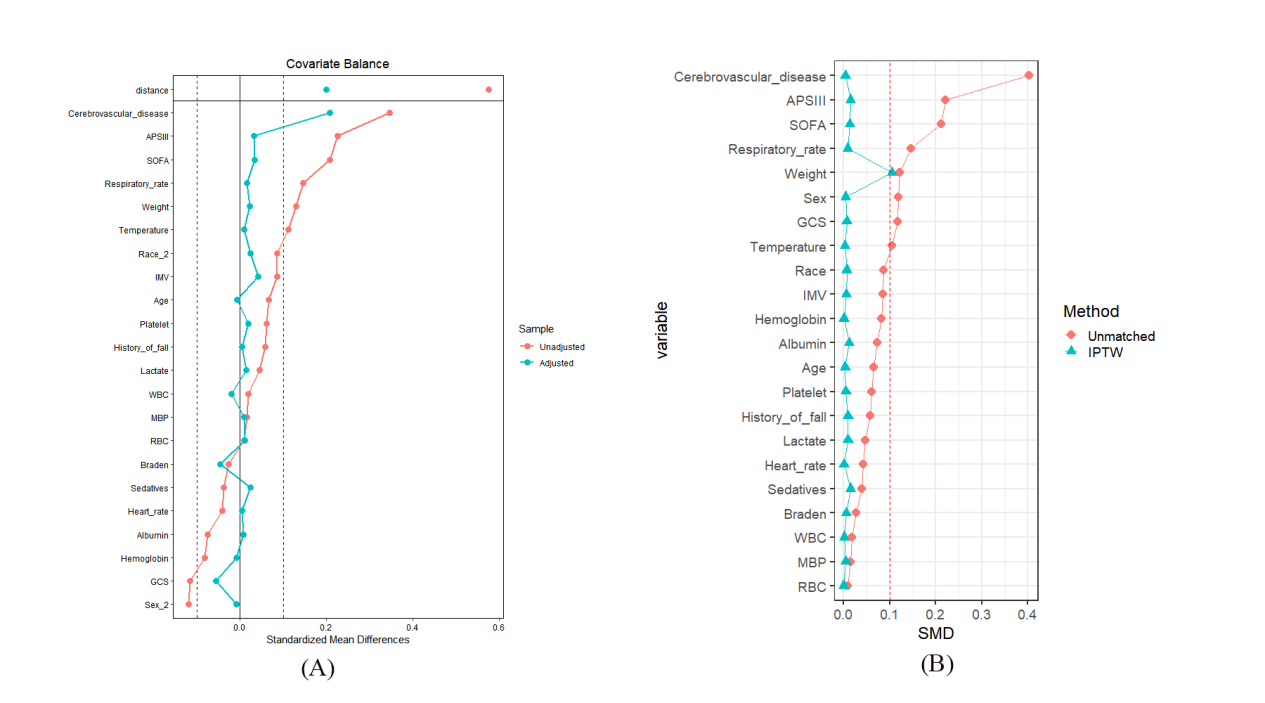


**Supplementary Figure 2**: Covariate balance before and after propensity score matching and inverse probability of treatment weighting

(A) Covariate balance before and after PSM

(B) Covariate balance before and after IPTW

Abbreviations: SMD, Standardized Mean Differences; PSM, Propensity Score Matching; IPTW, Inverse Probability of Treatment Weighting; APSIII, Acute Physiology Score III; SOFA, Sequential Organ Failure Assessment; IMV, Invasive Mechanical Ventilation; WBC, White Blood Cell count; MBP, Mean Blood Pressure; RBC, Red Blood Cell count; GCS, Glasgow Coma Scale.

Note: This figure shows the covariate balance for several clinical variables before and after applying propensity score matching and inverse probability of treatment weighting. The standardized mean differences are used to assess the balance between the treatment groups.
